# Identification of meat adulteration in minced meat samples labeled as beef and mutton in Tehran stores using duplex PCR

**DOI:** 10.1002/fsn3.4351

**Published:** 2024-07-28

**Authors:** Mohammad Doroudian, Mahdieh Soezi, Milad Rasouli, Maryam Arshadi Far, Maryam Yousefi Dehbidi, Pedram Maafi, Forough Yousefi, Mohammad‐Reza Ajouri, Bijan Omidi

**Affiliations:** ^1^ Department of Cell and Molecular Sciences, Faculty of Biological Sciences Kharazmi University Tehran Iran; ^2^ Infection Disease Research Center AJA University of Medical Sciences Tehran Iran; ^3^ Medical Biotechnology Research Center AJA University of Medical Sciences Tehran Iran; ^4^ Endocrinology and Metabolism Research Center Tehran University of Medical Sciences Tehran Iran; ^5^ Department of Physics Kharazmi University Tehran Iran; ^6^ Azmoon Salamat ASA Tehran Iran; ^7^ Department of Microbiology and Parasitology, School of Medicine Bushehr University of Medical Sciences Bushehr Iran; ^8^ The Persian Gulf Tropical Medicine Research Center, The Persian Gulf Biomedical Sciences Research Institute Bushehr University of Medical Sciences Bushehr Iran

**Keywords:** duplex PCR, meat, meat adulteration, meat products

## Abstract

Food fraud and profiteering are becoming increasingly common in the meat industry. Therefore, it is essential to identify such practices to prevent consumer deception and maintain food safety. This study aimed to determine the contents of minced meat samples labeled as beef and mutton in retail stores across Tehran province, Iran, to identify instances of meat adulteration. To this end, this study randomly collected 300 minced meat samples labeled as beef and mutton from Tehran stores over 4 years (2018–2022) and analyzed them using duplex polymerase chain reaction (PCR). The results revealed that more than 95% of the samples only contained beef, while only 5% of the samples matched the label and contained a mixture of beef and mutton. This discrepancy between the label and actual contents could be attributed to the price difference between beef and mutton, providing a financial incentive for producers to maximize profits. Given the potential for meat adulteration, increased monitoring of meat products is necessary, including detailed tests such as PCR, which is a fast, easy, sensitive, specific, and highly effective method for detecting meat adulteration. The findings of this study can assist in developing effective strategies to prevent meat adulteration and maintain consumer confidence in the meat industry.

## Graphical abstract

In this study, we conducted a comprehensive analysis of 300 minced meat samples labeled as beef and mutton, collected randomly from Tehran stores over a span of 4 years (2018–2022). Employing duplex polymerase chaiin reaction (PCR) techniques, we meticulously examined the meat samples, aiming to compare their actual contents with the information provided on the labels. Our findings shed light on a significant discrepancy between the labeled and actual meat contents, with over 95% of the samples containing only beef, despite being labeled as a mixture of beef and mutton. This revelation underscores the pervasive nature of meat adulteration in the Tehran market, driven primarily by economic incentives to maximize profits.

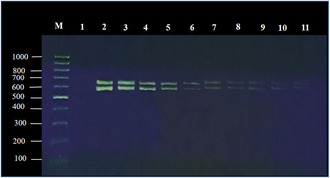

## 1 INTRODUCTION

Due to the increasing incidence of food fraud and the significant role that meat plays as a primary food source for humans, as well as for economic, legal, religious, safety, and health reasons, there is a pressing need for an effective method to investigate the extent of meat adulteration (Daniel et al., 2011; Li et al., 2020). On the other hand, for many people, eliminating meat from their diet is not pleasant because meat and meat products are important sources of essential nutrients, such as proteins, fats, minerals, and vitamins (De Smet, 2012; Higuera et al., 2019; Wyness, 2016). Considering that the original sources of raw materials in meat products are rendered unrecognizable postmincing, it becomes imperative to scrutinize the composition of ground meat. In light of numerous global reports on meat adulteration, this issue has surfaced as a significant worldwide concern (Ballin et al., 2009). A notable instance of deception in the meat industry is the 2013 European scandal, where products labeled as beef were found to contain horse meat (Zdeňková et al., 2018). Previous studies on meat products in Iran have revealed instances of false labeling and noncompliance of the labeled food content with the actual product (Farshidi et al., 2020; Mehdizadeh et al., 2014; Sarab, 2020). This includes the substitution of expensive and high‐quality meat with a less valuable or different kind (Darban Maghami et al., 2021; Doosti et al., 2014; Mousavi et al., 2015).

In Iran, dietary patterns exhibit a marked preference for meat, particularly beef and mutton, which are among the primary sources of protein consumed. Mutton is highly valued for its flavor and is often used in traditional Iranian dishes, such as kebabs, stews, and rice dishes. Beef is also popular and is used in various culinary preparations. However, these meats have notable differences in consumer preferences and market prices. In the Iranian market, mutton commands a higher price point compared to beef. This disparity in cost can be attributed to a multitude of factors, notably, the elevated expenses associated with sheep husbandry relative to cattle, coupled with a cultural predilection for mutton within specific traditional cuisines. Consequently, mutton's status as a luxury commodity and its consequent premium pricing structure create economic motivations for suppliers and merchants to engage in the adulteration of mutton with the less expensive beef as a means to augment profit margins (Doosti et al., 2014; Doroudian et al., 2023). This economic incentive is a significant driver of meat adulteration practices in the market. Consumer preferences in Iran are influenced by cultural, religious, and culinary factors (Doosti et al., 2014; Falahi et al., 2012). Many consumers prefer mutton for its distinct taste and its use in traditional dishes. However, some consumers may choose beef or mixed meat products as a more affordable alternative due to their higher prices (Roudsari et al., 2017). This creates a market environment where the accurate labeling of meat products is crucial for consumer trust and satisfaction.

Various analytical methods based on protein and DNA analysis can be used to identify the type of meat used in meat products (Barai et al., 1992; Montowska & Pospiech, 2010). Enzyme analysis, electrophoresis, and chromatography are among the protein analysis methods that may be used in conjunction with mass spectrometry to identify the type of meat used in meat products (Flores‐Munguia et al., 2000; Hsieh et al., 1996; Leitner et al., 2006). Molecular methods based on DNA can be used to identify the type of meat and may include techniques, such as single‐strand conformation polymorphism (SSCP), random amplified polymorphic DNA (RAPD), restriction fragment length polymorphism (RFLP), real‐time polymerase chain reaction (PCR), multiplex PCR, and simplex PCR (Ali et al., 2015; Dai et al., 2015; Li et al., 2019). Recent advancements in food authentication technologies have significantly enhanced the ability to detect and prevent food adulteration, ensuring food safety and integrity. For instance, DNAFoil technology offers a rapid and reliable method for identifying adulterated food products, addressing growing concerns over food fraud and consumer deception (El Sheikha, 2019). Similarly, efficient DNA extraction methods have improved the accuracy and reliability of meat authentication processes, enabling the precise identification of meat species in various products (Khairil Mokhtar et al., 2020). Furthermore, DNA‐based authentication technologies have emerged as powerful tools for verifying the compliance of meat products, particularly halal meats, with religious dietary laws, offering high sensitivity and specificity in detecting meat adulteration (El Sheikha et al., 2017). Implementing these techniques guarantees a comprehensive assessment of meat characteristics throughout various stages, including meat processing, packaging, transportation, storage, and marketing. By employing these methods, the quality and safety of meat products can be thoroughly evaluated, ensuring consumer satisfaction and promoting industry standards.

Molecular techniques, particularly PCR, are commonly used for various detection purposes (Cai et al., 2021; Chaudhary & Kumar, 2022; Doroudian et al., 2023). They have also been employed to determine the type of meat due to their high sensitivity, accuracy, and cost‐effectiveness (Barai et al., 1992; Li et al., 2020). Duplex and multiplex PCRs are highly sensitive and accurate methods for identifying meat content. They can also accelerate the testing process and reduce costs compared to species‐specific PCR. Unlike species‐specific PCR, which requires a separate reaction tube for each animal species, multiplex PCR can test multiple species in a single reaction tube and can work with very small sample amounts (Rezazadeh et al., 2014). Performing multiplex PCR testing requires accurate determination of important parameters for each test. These include the approximate concentration of primers at different loci, PCR buffer concentration, suitable temperature of each cycle, and equilibrium between magnesium chloride and deoxynucleotide concentrations (Hao et al., 2010; Mehdizadeh et al., 2014). The electrophoresis gel is commonly used to study the results of PCs, which separate DNA fragments based on their molecular weight (Sarab, 2020). Table 1 compares the duplex PCR method with other methods documented in the literature, including PCR, enzyme‐linked immunosorbent assay (ELISA), and DNA barcoding.

**TABLE 1:** Comparison of different methods for meat species identification.

Method	Advantages	Disadvantages	References
Duplex PCR	Can identify multiple meat species simultaneously. Cost‐effective and time‐saving. High specificity with the use of specific primers. Can detect less than eight animal origins in a one‐tube reaction platform.	Technological challenges due to mutual interference of components. Less than eight meat species can be discriminated in one reaction platform.	Cai et al. (2021); Cheng et al. (2022); Qin et al. (2019); and Yang et al. (2022)
PCR	Reliable for meat species identification. It can be used for rapid detection.	It may contain residual solvents, such as chloroform and phenol, leading to the inhibition of PCR amplification.	Chaudhary and Kumar (2022) and Doosti et al. (2014)
ELISA	Can successfully identify beef in analysis.	Less successful in identifying pork in assays compared to real‐time PCR.	Aprilia et al. 2022; Perestam et al. (2017); and Yörük (2021)
DNA barcoding	Promising for the sequencing‐based identification of meat and poultry species in food products.	DNA degradation during processing can be a challenge.	Adenuga and Montowska (2023) and Hellberg et al. (2017)

Despite the availability of various analytical methods, there remains a significant gap in the comprehensive and systematic analysis of minced meat products in Tehran over an extended period. Previous studies have often focused on specific instances or limited sample sizes, lacking a broader temporal and geographical scope. This study addresses this gap by providing a focused analysis of 300 minced meat samples collected from Tehran stores over 4 years (2018–2022). The use of duplex PCR allows for the simultaneous detection of beef and mutton in a single reaction, enhancing the efficiency and accuracy of meat adulteration detection. Our findings reveal a significant discrepancy between the labels and actual meat contents, highlighting a prevalent issue of meat adulteration driven by economic factors. This study contributes to the existing body of knowledge and has practical implications for developing effective strategies to combat meat adulteration and uphold consumer trust in the meat industry.

## 2 MATERIALS AND METHODS

### 2.1 Sample preparation

Three hundred samples of raw minced meat from different brands in Tehran province were randomly collected from eight suppliers over 4 years. The samples were collected to ensure a representative picture of the Tehran market. We specifically targeted various retail stores across the city. Each sample was a prepackaged product purchased directly from the store shelves in its original form. To confirm the integrity of the product, we inspected the packaging for any signs of tampering. The labels on the packages provided information on the composition, indicating that all the samples were a mixture of beef and mutton. It is important to note that all the collected samples were prepackaged with the composition information readily available on the label. The samples were kept at −20°C until DNA extraction to prevent enzymatic degradation. Pure beef and mutton samples were also obtained as positive control from livestock centers.

### 2.2 DNA extraction

A total of 200 mg of each sample and control were placed into a microtube, and DNA extraction and purification were carried out using the DNeasy mericon Food Kit (Qiagen, Germany, Cat No. 69514) with a spin column method. The DNA purification process adhered to the guidelines outlined in the extraction kit. Initially, 1 μL of lysis buffer and 3 μL of proteinase K were introduced to the samples' microtubes. These microtubes were then subjected to an incubation period at 60°C for 30 min. Following this, the samples underwent centrifugation at 4500 rpm (revolutions per minute) for 5 min, and 700 μL of the supernatant was combined with 500 μL of chloroform (Sigma, Cat No. C2432). After another centrifugation step at 14,000 rpm for 15 min, 350 μL of the supernatant was cautiously transferred to a fresh microtube, and 350 μL of binding buffer was added to each sample.

The next step involved transferring the samples to a DNA purification column and centrifuging them at 14,000 rpm for 1 min. After the washing process, 100 μL of elution buffer was added to each column and centrifuged at 14,000 rpm for 1 min. The purified DNA was then collected in new microtubes. Subsequently, the purity and concentration of the DNA were determined using a NanoDrop device (Thermo Scientific™, NanoDrop 2000) at a wavelength of 260–280 nm. An absorption rate of 1.6–2 was recorded, indicating high DNA purity. The DNA samples were prepared at a concentration of 100 nanograms per microliter (ng/μL) and stored at −20°C until PCR was conducted.

### 2.3 PCR

**FIGURE 1:**
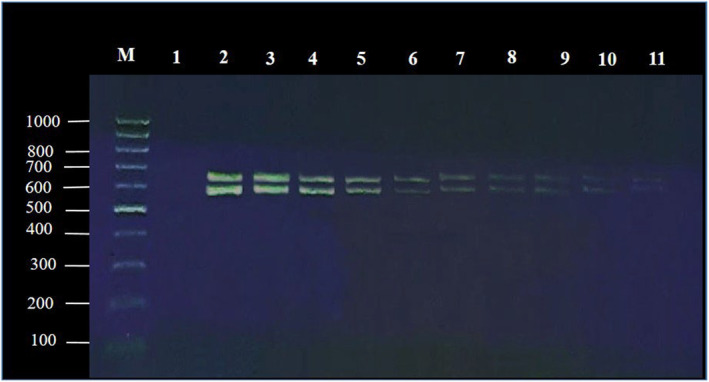
Limit of detection of duplex PCR assays of pure sheep (664 bp) and cattle (537 bp) tissue DNAs. M: 100 bp DNA ladder. Lane 1: The negative control. Lanes 2–12: PCR for the detection of samples. Lanes 2 and 3: 10 ng/μL. Lanes 4 and 5: 1 ng/μL. Lanes 6 and 7: 0.1 ng/μL. Lanes 8 and 9: 0.01 ng/μL. Lanes 10 and 11: 0.001 ng/μL.

For the duplex PCR system, 1 μL (50 ng) of DNA from each sample was combined with 10 pmol (picomoles) of the forward primer and 10 pmol of the reverse primers. Additionally, 25 μL of PCR Master Mix (Qiagen, Germany, Cat No. 206143) was included. The final volume of the reaction mixture was adjusted to 50 μL with double‐distilled water (DDW). The PCR conditions comprised an initial denaturation step at 95°C for 10 min, followed by 35 cycles of denaturation at 95°C for 30 s, annealing at 60°C for 30 s, and elongation at 72°C for 30 s. A final elongation step was conducted at 72°C for 7 min. The PCR products were then analyzed under ultraviolet (UV) light (Bio‐Rad, USA) after electrophoresis using a 2% agarose gel (Thermo Fisher Scientific, USA) containing 0.5 μg/mL ethidium bromide (Sigma, USA). The developed assay was validated to confirm its specificity, sensitivity, reliability, and reproducibility (Park et al., 2013). The primer pairs used in this study are shown in Table 2. Additionally, the DNA concentration of meat samples was aseptically diluted five times, ranging from 10 ng/μL to 0.001 ng/μL, to serve as a template for the duplex PCR assay. This was performed to evaluate the diagnostic sensitivity of the duplex PCR system. The PCR method demonstrated a detection limit of at least 0.001 ng/μL genomic DNA (Figure 1).

**TABLE 2:** Primer pairs used in this study.

Gene target	Primer name	Primer sequence 5′ → 3′	Amplified fragment (bp)	Annealing temperature	Ref
Cytochrome b	Cattle (reverse primer)	TAGTAGGTGGACTATGGCAATT	537	60°C	Park et al. (2013)
Cytochrome b	Sheep (reverse primer)	GCATGAGGATGAGGATTAGTAGGATAGCA	664	60°C	Park et al. (2013)
Cytochrome b	MIF (common forward primer)	GACCTCCCAGCCCCATCAAACATCTC ATCATGATGAAA	–	60°C	Park et al. (2013)

### 2.4 Sensitivity of the duplex PCR assay

To ensure the robustness and reliability of the duplex PCR assay, we conducted a sensitivity test using DNA extracted from pure beef and mutton samples. The dilution process entailed diluting the DNA five times, spanning from an initial concentration of 10 ng/μL to a final concentration of 0.001 ng/μL using sterile double‐distilled water (DDW). To assess the sensitivity of the duplex PCR, duplicate evaluations were performed under optimized reaction conditions. Ultimately, 1 μL (50 ng) of each dilution was used as a template for the duplex PCR analysis (Hou et al., 2015).

## 3 RESULTS AND DISCUSSION

**FIGURE 2:**
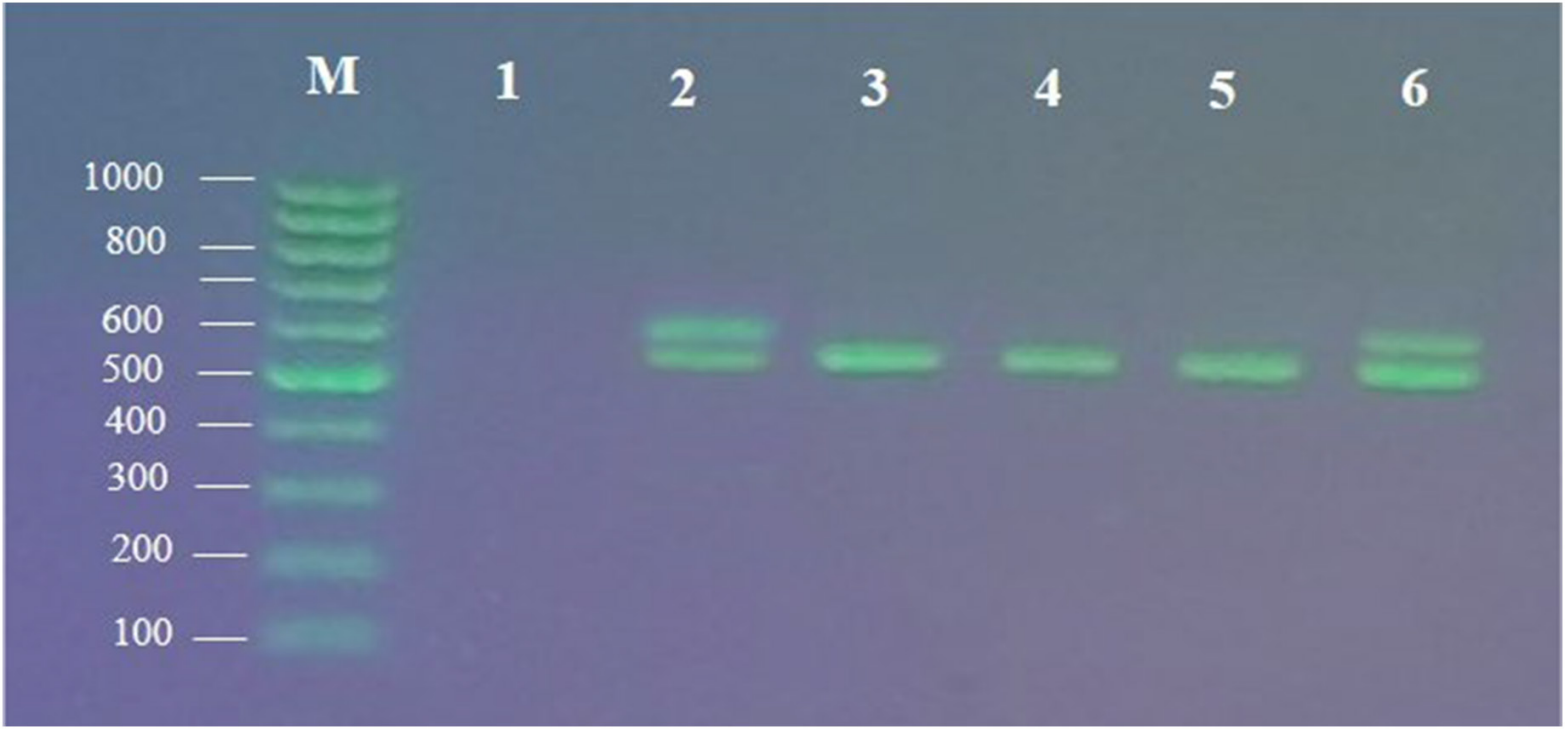
The result of agarose gel electrophoresis of duplex PCR products. M: 100 bp DNA ladder. Lane 1: The negative control. Lane 2: Duplex PCR of sheep (664 bp) and cattle (537 bp) tissue DNAs as a positive control. Lane 3–6: Duplex PCR products of meat samples with primers designed for Lanes 1 and 2.

This study employed duplex PCR as a suitable and cost‐effective method for detecting meat adulteration in 300 randomly collected minced meat samples in Tehran, Iran. The primers were designed to target the mitochondrial cytochrome b gene, amplifying 537 bp and 664 bp fragments for beef and mutton, respectively. No cross‐contamination, inhibition, or unexpected PCR products were observed after analyzing the electrophoresis pattern. The primers yielded the anticipated fragments solely in the presence of the respective DNA, confirming the assay's specificity to the target species and ensuring no misidentification of other meat species. Mitochondrial gene nucleotide sequences were utilized to distinguish between various types of meat, and the duplex PCR assay was fine‐tuned for the simultaneous identification of multiple species using control samples. The presence of a positive control band confirmed the specificity of the bands obtained from the designed primers. The PCR products derived from the DNA‐extracted species showed no cross‐reactivity with DNA from other species, affirming the assay's specificity. The results showed that more than 95% of the samples contained only beef, which was unexpected since the product labels indicated a 50–50 or similar percentage of beef and mutton (Table 3). The inconsistency between the product contents and labels, along with the presence of cheaper beef, confirmed the occurrence of meat adulteration in more than 95% of products. The result of duplex PCR products from a meat sample was displayed as representative (Figure 2).

**TABLE 3:** Summary of our findings.

Market type	Sample origin	Number of samples	Label indicated beef and mutton	Actual content (beef only)	Actual content (beef and mutton)	Adulteration rate (%)
Supermarket	North Tehran	50	50	48	2	96
Supermarket	South Tehran	50	50	47	3	94
Butcher shop	East Tehran	50	50	49	1	98
Butcher shop	West Tehran	50	50	48	2	96
Local market	Central Tehran	50	50	47	3	94
Local market	Suburban area	50	50	46	4	92
Total	All regions	300	300	285	15	95

Meat has been a target for adulterators for decades because of its high commercial value, and reports on meat adulteration have surfaced worldwide. There is a continued need to develop sensitive and reliable methods for identifying meat species, driven by both economic and religious factors. Each technique used to detect meat adulteration has its own advantages and disadvantages, and therefore, the selection of a method should be based on the specific application and requirements. Isoelectric focusing is a common technique used for identifying species in cooked meat. Electrophoresis has the disadvantage of requiring several hours and presenting low reproducibility. Liquid chromatography is another method that can be used, but it is expensive and relatively slow. The immunodiffusion gel technique is a reproducible method that is often used for screening noncooked foods and routine analysis. On the other hand, the ELISA technique is a protein‐based method that can not only be useful for detecting specific molecules, but may also produce false results or cross‐reactivity (Barai et al., 1992; Flores‐Munguia et al., 2000; Li et al., 2020; Zahran & Hagag, 2015). Food safety monitoring agencies require highly sensitive, accurate, and inexpensive techniques. Duplex PCR is one such DNA‐based method that has been used in recent years to detect meat authentication. Its accuracy, speed, and sensitivity make it an attractive option for food safety monitoring agencies (Li & Guan, 2019).

Hassanzadeh et al. (2018) reported that seven samples were contaminated with donkey meat in Tabriz, Iran. A study conducted by Darban Maghami et al. (2021) in Mashhad, Iran, confirmed that minced red meat samples were adulterated by the addition of gizzard and chicken skin in addition to skeletal muscle, smooth muscle, and adipose tissue. Another study in Iran found that chicken meat was detected in 47.2% of ground meat samples, while donkey meat was present in 0.7% of samples (Mousavi et al., 2015). Our results, coupled with these studies, indicate that meat adulteration practices are common in Iran, motivated for the most part by the economically encouraged replacement of higher‐cost meats with lower‐cost alternatives, such as donkey, chicken, and offal (Doosti et al., 2014). In Iran, the government has established regulations to ensure the authenticity and safety of meat products. The Iranian National Standards Organization (INSO) has set specific standards for meat labeling and composition to prevent adulteration and protect consumers. However, the high prevalence of meat adulteration observed in our study indicates a gap between regulatory standards and actual market practices (Doroudian et al., 2023). The widespread discrepancy observed across Iran, where labeling fails to reflect content, necessitates stricter enforcement of regulations and routine testing to guarantee compliance and protect consumers (Doosti et al., 2014; Doroudian et al., 2023).

It should be highlighted that the issue of meat adulteration is not limited to Iran but has been reported globally. The 2013 horse meat scandal in Europe, where horse meat was substituted for beef in various processed meat products, brought international attention to the widespread nature of meat adulteration and the need for robust authentication methods (Zdeňková et al., 2018). Similarly, another study compared ELISA and PCR assays for detecting pork adulteration in halal‐labeled beef products, underscoring the importance of accurate detection methods for ensuring compliance with dietary laws and consumer preferences (Aprilia et al., 2022). These global incidents, along with our findings, underscore the economic motivations driving meat adulteration practices, where cheaper or undesirable meats are substituted for more expensive or preferred varieties to maximize profits, often at the expense of consumer trust and food safety (Moyer et al., 2017).

While our study and several others have reported high rates of meat adulteration, it is important to acknowledge that the prevalence and types of adulteration can vary across different regions and contexts. Some studies have reported lower rates of adulteration due to stricter regulatory enforcement, better industry practices, or different economic incentives (Ehmke et al., 2019; Haji et al., 2023). Additionally, the types of adulteration may differ based on local dietary preferences and economic factors, such as the use of pork to adulterate beef products in regions where pork is cheaper than beef (Gecaj et al., 2021; Yang et al., 2022). These contradictory findings highlight the importance of considering regional and cultural factors when addressing meat adulteration, as well as the need for tailored strategies and regulations to combat specific forms of adulteration prevalent in different contexts (Yang et al., 2022). The findings of this study have significant implications for food safety and regulation. The high prevalence of meat adulteration in Tehran stores underscores the need for increased monitoring and enforcement of labeling regulations. Implementing routine testing using sensitive and specific methods like duplex PCR can help detect and prevent fraudulent practices, thereby ensuring the integrity of meat products and protecting consumer interests. Similar recommendations have been made in other studies, highlighting the importance of robust analytical methods for food authentication (Skouridou et al., 2019; Yang et al., 2022).

## 4 CONCLUSIONS

This study aimed to identify meat adulteration in minced meat samples labeled as beef and mutton in Tehran stores using duplex PCR. Our findings revealed that more than 95% of the samples contained only beef, while only 5% matched the label and contained a mixture of beef and mutton. This significant discrepancy between the label and actual contents highlights a prevalent issue of meat adulteration driven by economic incentives. Our findings are important because of their implications for food safety and consumer protection. The high prevalence of meat adulteration in Tehran stores reveals the need for increased monitoring and stringent enforcement of labeling regulations. Implementing routine testing using sensitive and specific methods like duplex PCR can help detect and prevent fraudulent practices, ensuring the integrity of meat products and maintaining consumer trust. Overall, this study provides valuable insights into the extent of meat adulteration in Tehran stores and highlights the effectiveness of duplex PCR as a detection method. These findings can inform the development of effective strategies to combat meat adulteration and uphold food safety standards, ultimately benefiting consumers and the meat industry.

## AUTHOR CONTRIBUTIONS

**Mohammad Doroudian:** Conceptualization (equal); formal analysis (equal); funding acquisition (equal); methodology (equal); project administration (equal); supervision (equal); writing – original draft (equal). **Mahdieh Soezi:** Formal analysis (equal); investigation (equal); methodology (equal); resources (equal); validation (equal); writing – original draft (equal). **Milad Rasouli:** Investigation (equal); methodology (equal); writing – original draft (equal); writing – review and editing (equal). **Maryam Arshadi Far:** Data curation (equal); formal analysis (equal); investigation (equal); methodology (equal). **Maryam Yousefi Dehbidi:** Investigation (equal); methodology (equal); software (equal). **Pedram Maafi:** Investigation (equal); methodology (equal); software (equal). **Forough Yousefi:** Data curation (equal); investigation (equal); supervision (equal); validation (equal). **Mohammad‐Reza Ajouri:** Investigation (equal); methodology (equal); resources (equal); visualization (equal). **Bijan Omidi:** Data curation (equal); methodology (equal); project administration (equal); resources (equal); supervision (equal); validation (equal).

## CONFLICT OF INTEREST STATEMENT

The authors declare no conflicts of interest.

## Data Availability

The data are available from the corresponding author upon reasonable request.
